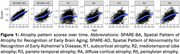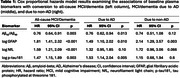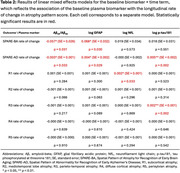# Association of plasma biomarkers with longitudinal change in age‐ and Alzheimer’s disease‐related brain atrophy patterns

**DOI:** 10.1002/alz70861_108568

**Published:** 2025-12-23

**Authors:** Murat Bilgel, Ishaan Shah, Jasmine Cooper, Yang An, Keenan A. Walker, Abhay Moghekar, Zhijian Yang, Christos Davatzikos, Susan M. Resnick

**Affiliations:** ^1^ National Institute on Aging, National Institutes of Health, Baltimore, MD USA; ^2^ University of Michigan, Ann Arbor, MI USA; ^3^ Johns Hopkins University School of Medicine, Baltimore, MD USA; ^4^ University of Pennsylvania, Philadelphia, PA USA

## Abstract

**Background:**

Demonstrating the specificity of plasma biomarkers to AD‐related neurodegeneration would add support to their prognostic and diagnostic clinical use.

**Method:**

Participants from the Baltimore Longitudinal Study of Aging were cognitively unimpaired at the time of their plasma Aβ_42_/Aβ_40_, GFAP, NfL (Quanterix Neurology 4‐plex E [N4PE]; *N*=818 participants), and *p* ‐tau181 (Quanterix pTau‐181 V2; *N*=556 participants) measurements. Longitudinal 3 T MRIs (2,295 and 1,802 visits for the N4PE and *p* ‐tau181 analyses, respectively) were used to quantify multidimensional brain atrophy pattern scores (Figure 1), including Spatial Pattern of Abnormality for Recognition of Early Brain Aging (SPARE‐BA) and Alzheimer’s disease (SPARE‐AD) scores, which are individualized indices of age‐ and AD‐related brain atrophy, respectively. We also quantified R‐indices describing five dominant dimensions of brain aging: subcortical atrophy (R1), mediotemporal lobe atrophy (R2), parieto‐temporal atrophy (R3), diffuse cortical atrophy (R4), and perisylvian atrophy (R5). We examined the associations of plasma biomarkers with conversion to a clinical diagnosis of mild cognitive impairment (MCI)/dementia due to AD using Cox proportional hazards models, and with longitudinal change in atrophy pattern scores using linear mixed effects models. Models were adjusted for baseline age (and age^2^ for linear mixed effects models), sex, age × sex, race, years of education, and estimated glomerular filtration rate.

**Result:**

Higher plasma *p* ‐tau181 was associated with a higher risk of conversion to MCI/dementia due to AD (HR = 1.5, 95% CI = [1.1, 2], *p* = 0.0045) (Table 1) and steeper increases in AD‐related atrophy patterns (SPARE‐AD estimate = 0.005, SE = 0.0016, *p* = 0.0017), specifically in parieto‐temporal areas (R3 estimate = 0.0022, SE = 0.00071, *p* = 0.002) (Table 2). Lower Aβ_42_/Aβ_40_ and higher GFAP were also associated with a higher risk of conversion to MCI/dementia due to AD and steeper increases in SPARE‐AD, but their associations were not limited to AD‐related neurodegeneration: they were also associated with steeper longitudinal changes in SPARE‐BA.

**Conclusion:**

Among cognitively unimpaired individuals, plasma *p* ‐tau181 exhibits specificity to the risk of conversion to MCI/dementia due to AD and longitudinal change in AD‐related neurodegeneration, specifically, parieto‐temporal atrophy, whereas Aβ_42_/Aβ_40_ and GFAP are associated with both age‐ and AD‐related neurodegeneration.